# Effect of intravitreal dexamethasone on macular edema in von Hippel-Lindau disease assessed using swept-source optical coherence tomography: a case report

**DOI:** 10.1186/s13256-018-1787-8

**Published:** 2018-09-06

**Authors:** Angelo Maria Minnella, Valeria Pagliei, Martina Maceroni, Matteo Federici, Gloria Gambini, Aldo Caporossi

**Affiliations:** Institute of Ophthalmology, Università Cattolica del Sacro Cuore, Fondazione Policlinico Universitario A. Gemelli - IRCCS, Rome, Italy

**Keywords:** Retinal hemangioblastoma, Von Hippel-Lindau disease, Swept-source OCT, *En face* OCT, Macular edema, Innovative biotechnology

## Abstract

**Background:**

Von Hippel-Lindau disease is a rare hereditary syndrome caused by germinal mutations in a von Hippel-Lindau tumor-suppressing gene. Retinal hemangioblastoma is the ocular hallmark lesion of von Hippel-Lindau disease.

**Case presentation:**

A 20-year-old Caucasian woman presented to our institution with painless visual impairment in the right eye. A fundus ophthalmoscopic evaluation and swept-source optical coherence tomographic examination revealed a retinal hemangioblastoma associated with cystoid macular edema. On the basis of the clinical ocular findings and genetic analysis, von Hippel-Lindau disease was diagnosed. Following an intravitreal injection of ranibizumab, off-label administration of intravitreal dexamethasone was considered to reduce the edema. An almost complete resolution of the edema in the macular area was observed 1 week after the injection. Finally, laser photocoagulation and transconjunctival cryotherapy were performed; the patient developed “ablatio fugax” after cryotherapy.

**Conclusions:**

In our experience, intravitreal dexamethasone administration has proven to be a useful tool for reducing retinal hemangioblastoma-related macular edema in von Hippel-Lindau disease and may be considered a potentially valuable treatment that can be used in combination with other therapies.

## Background

Von Hippel-Lindau (VHL) disease is an autosomal dominantly inherited multisystem cancer caused by germline variation in the *VHL* gene on chromosome 3p25p26 [1]. Some patients affected by VHL disease (49–85%) develop retinal hemangioblastoma (RHB) [2, 3], and, despite its benign nature, RHB may cause several sight-threatening complications, such as macular exudation, retinal traction, retinal detachment, retinal and vitreous hemorrhage, and neovascular glaucoma [4]. We describe the effect of intravitreally administered dexamethasone on macular edema secondary to RHB using swept-source optical coherence tomography (SS-OCT).

## Case presentation

**Fig. 1:**
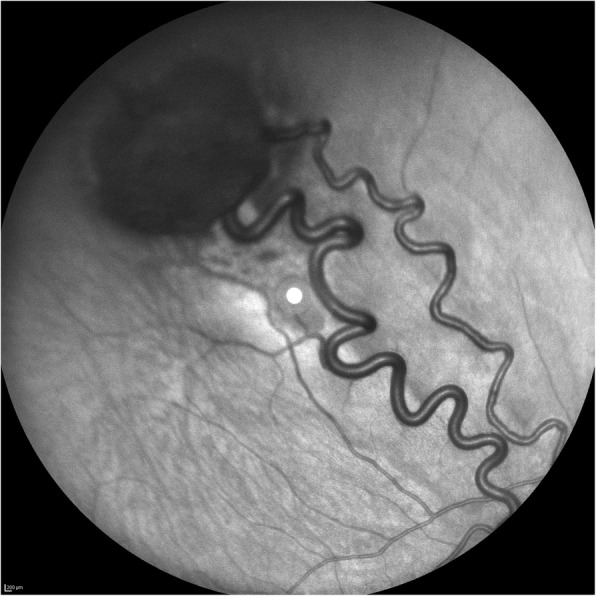
Infrared image of the right eye (RE). Retinal hemangioblastoma with prominent feeder vessels in the superior temporal region of the retina in the RE

A 20-year-old Caucasian woman presented to our institution with decreased vision in the right eye (RE) with a 4-month evolution**.** The patient’s vital signs were within normal limits, and no abnormalities were noticed upon physical and neurological examination. Similarly, her past medical history was unremarkable. Her best corrected visual acuity (BCVA) was 50 ETDRS (Early Treatment Diabetic Retinopathy Study) letters in the RE and 84 ETDRS letters in the left eye. Her intraocular pressure (IOP) was normal in both eyes, and the result of her anterior segment examination was unremarkable. Upon ophthalmoscopic examination, a red-colored globular lesion of 3 disk diameters (DDs) with prominent feeder vessels was noticed in the superior temporal region of the retina in the RE (Fig. 1). The fellow eye was normal on fundus examination, and no other lesions were found in the posterior pole or periphery. Optical coherence tomography (OCT) was carried out using the DRI OCT Triton™ SS-OCT device (Topcon Medical, Tokyo, Japan).

**Fig. 2:**
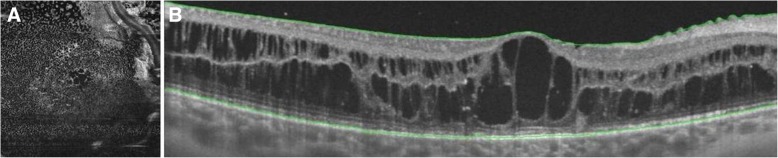
Structural optical coherence tomographic *en face* scan (**a**) and B-scan (**b**) of the right eye. The cystoid edema involves the inner and outer nuclear layers of the posterior pole, extending outside the vascular arcades

A structural OCT B-scan of the RE showed a diffuse cystoid macular edema (central macular thickness of 450 μm) (Fig. 2b). The *en face* scan enabled assessment of the extent of the cystoid edema, which involved the posterior pole and expanded outside the vascular arcades (Fig. 2a).

The patient’s family history was collected, and it was discovered that the patient’s mother had died of pulmonary edema during pregnancy at the age of 40. A genetic test was carried out, which showed a variant in the *VHL* gene: c.335A>G(p.Y112C). In our patient, this variant was in the heterozygous state. On the basis of genetic findings and considering the presence of RHB, the diagnosis of VHL disease was made. A systemic study was conducted to search for other organs with lesions. Results of renal ultrasonography and magnetic resonance imaging of the brain were both negative for visceral lesions.

**Fig. 3:**
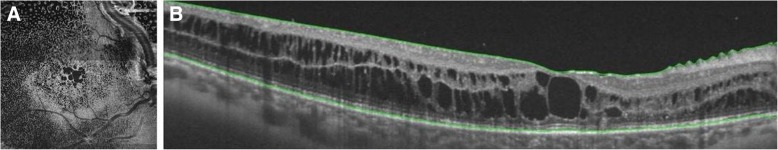
**a** and **b** Structural optical coherence tomographic (OCT) scan of the right eye 40 days after intravitreal injection of ranibizumab. A reduction in the number and size of the cystic spaces and in the central macular thickness can be observed on the OCT B-scan (**b**). A decrease in the extension of cystoid edema is observed on the *en face* scan as well (**a**)

**Fig. 4:**
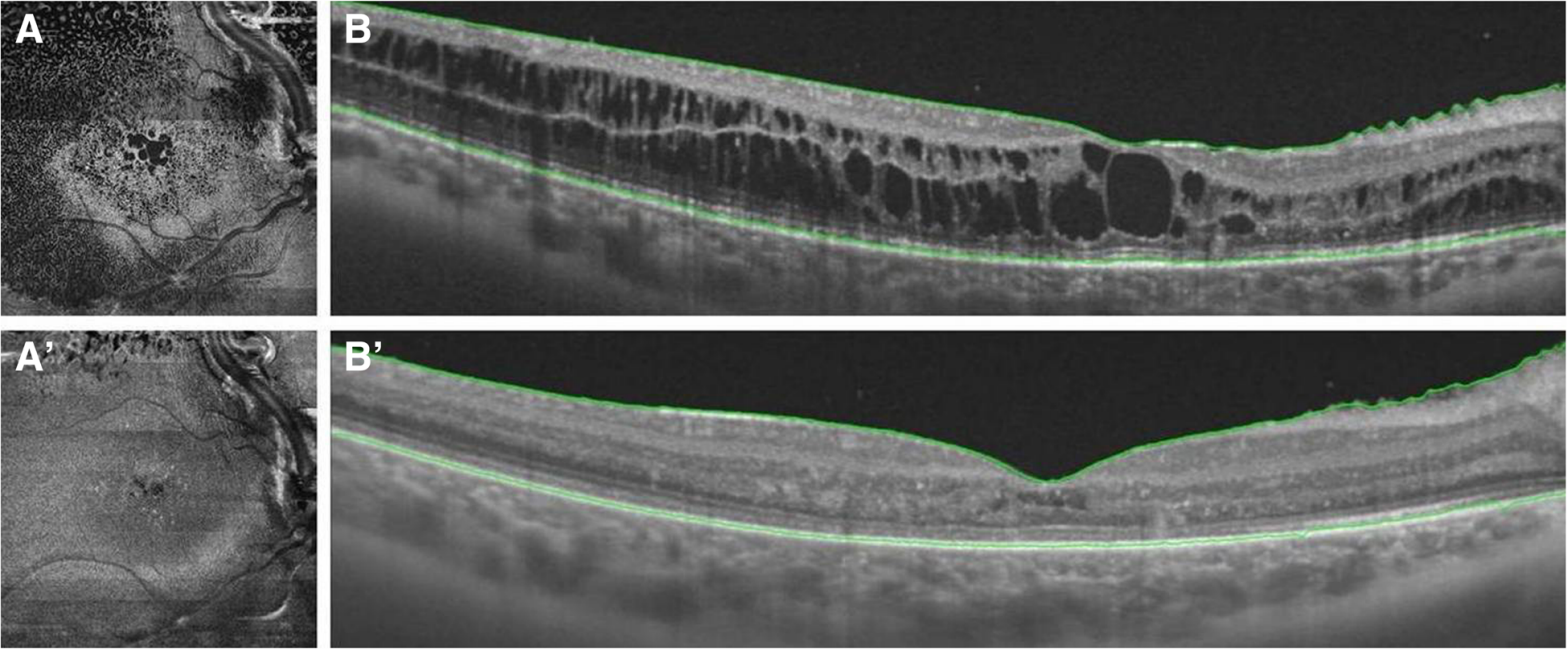
**a** and **b** Structural optical coherence tomographic (OCT) scans before and 1 week after the injection of slow-release intravitreal dexamethasone implant. Although an initial improvement is observed after the injection of ranibizumab, cystic spaces in both the inner and outer nuclear layers persist (**b**). Cystoid edema involves the posterior pole entirely, extending outside the vascular arcades (**a**). Structural OCT B-scan performed 1 week after intravitreal dexamethasone implant administration that shows the absence of cystic spaces, a considerable decrease in central macular thickness, and a restoration of retinal profile and foveal pit (**b′**). A significant reduction in the extension of the cystoid edema is observed on the *en face* scan, on which cysts appear to be located in a small area in the upper part of the image above the superior vascular arcade (**a′**)

The patient received an intravitreal injection of ranibizumab and was then followed every month. Forty days after the injection, an OCT B-scan and an OCT *en face* scan revealed a reduction in RE macular edema (Fig. 3a, b). The patient’s BCVA was 50 ETDRS letters in the RE. Considering the persistence of cystic spaces, an intravitreal injection of slow-release dexamethasone was considered to reduce macular edema in preparation for cryotherapy. At 60 days following ranibizumab administration, a slow-release intravitreal dexamethasone implant (IDI) was injected. A structural OCT B-scan performed 1 week after IDI showed the almost complete absence of cystic spaces in the subfoveal and perifoveal areas (Fig. 4b′) with complete restoration of the retinal profile (Fig. 4a′). Inconsistent with the improvement in retinal morphology, the patient complained of visual impairment and recurring headache. BCVA was found to be 42 ETDRS letters in the RE. No changes IOP values were observed during the follow-up examinations.

**Fig. 5:**
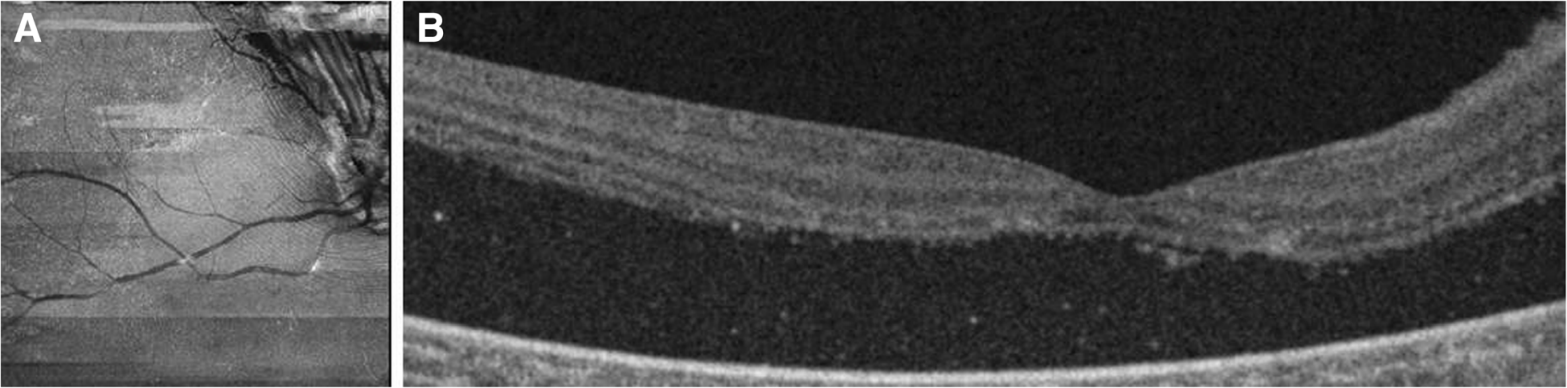
**a** and **b** Structural optical coherence tomographic (OCT) scans acquired 20 days after triple freeze-thaw technique transconjunctival cryotherapy. OCT B-scan (**b**) obtained 20 days after cryotherapy was performed shows a marked serous retinal detachment involving the macular region. A distortion of the posterior pole is observed on the *en face* scan (**a**)

Twenty days after IDI, the patient’s RHB was treated with a combination of laser photocoagulation and a triple freeze-thaw technique of transconjunctival cryotherapy. Although the patient’s retinal profile seemed to have been restored, OCT images obtained 20 days after cryotherapy was performed (Fig. 5) showed increased exudation causing a massive and wide serous retinal detachment. The patient was then followed every month for 6 months, and, considering the persistence of the exudative retinal detachment and having ruled out the presence of a retinal break, she is currently under evaluation for pars plana vitrectomy (possibly associated with lens extraction and/or scleral buckling) and endovitreal tumor treatment.

## Discussion

VHL disease is an inherited multisystem cancer that affects different organs, including the retina. Development of RHB represents the earliest and most common clinical sign and may lead to complications such as macular exudation, as observed in our patient. This report describes the off-label use of intravitreal dexamethasone, which may represent a novelty in the management of macular fluid secondary to RHB in VHL disease and may be considered a potentially valuable treatment that can be used in combination with other therapies.

Different ways of treating this ocular tumor have emerged, ranging from observation to laser photocoagulation [5], cryotherapy, plaque radiotherapy, photodynamic therapy [6], and vitreoretinal surgery [7]. In recent years, a further understanding of the mechanisms behind the development of tumors in VHL disease has resulted in wider use of vascular endothelial growth factor (VEGF)-targeted strategies. Tumorigenesis in VHL disease has been linked to somatic mutations or inactivation of the remaining wild-type VHL allele that affects the proper functioning of the VHL protein (pVHL). pVHL has been recognized as part of a multiprotein complex that is responsible for regulating hypoxia-inducible factor (HIF) destruction [8]. As a consequence of its loss of function, cells become unable to degrade HIF, whose excessive accumulation leads to the overexpression of hypoxia-induced genes such as VEGF, platelet-derived growth factor, transforming growth factor, and erythropoietin [9]. VEGF in particular induces angiogenesis [10] and is related to increased vascular permeability [11].

Considering the above-mentioned pathogenic process, several systemically delivered or intravitreally injected VEGF-targeted therapies have been tested. The effects of bevacizumab [12], ranibizumab [13], and the VEGF receptor inhibitor SU5416 [14], either alone or combined with other treatments, have already been described. These therapeutic strategies have proven to be valuable approaches with a positive impact, especially on macular edema rather than on the tumor.

IDI 0.7 mg (Ozurdex™; Allergan, Irvine, CA, USA) already represents a tool for the treatment of retinal vascular diseases such as diabetic macular edema [15] and retinal vein occlusion [16]. The use of dexamethasone in the treatment of cystoid edema related to VHL disease was taken into account, considering the activity of corticosteroids against the inflammatory mediators that are responsible for macular exudation [17]. In the case of VHL disease, these molecules are released as a consequence of the hypoxia-induced inflammatory status brought about by the dysregulation of HIF degradation.

The effect of Ozurdex™ in our patient was assessed by performing structural SS-OCT analysis, and both *en face* scans and B-scans were acquired. In our experience, dexamethasone has appeared to be effective in reducing macular thickness and restoring retinal profile and structure as seen upon OCT B-scan. On one hand, the *en face* scans of our patient provided a useful tool to investigate the changes in the extension of the cystoid edema, which appeared to be reduced after the administration of Ozurdex™. On the other hand, dexamethasone injection seemed to have no effect on the structure and size of the hemangioblastoma.

Unfortunately, cryotherapy was associated with increased exudation and serous retinal detachment (“ablatio fugax”). As demonstrated by several studies, large tumor size (> 2 DD) and the presence of massive exudation at diagnosis are associated with a poor response to treatment and poor visual outcome [18, 19]. Thus, the exudation and vision loss observed in our patient, despite IDI injection, could be regarded as a complication of cryotherapy whose development is imputable to the tumor features. Indeed, on one hand, the intraretinal fluid appears to have been absorbed after dexamethasone implant; on the other hand, the appearance of subretinal fluid and exudation after cryotherapy suggests that a different underlying pathogenetic mechanism of intraretinal fluid versus subretinal exudation should be considered, thus providing additional support for our hypothesis. The amount of exudation without IDI is hardly predictable, but according to our experience, the presence of steroids, especially when administered intravitreally, could decrease the exudation related to the tumor growth or the ablatio fugax after cryotherapy/laser tumor treatment.

## Conclusions

The aim of this report is to illustrate the off-label use of slow-release IDI in a patient with cystoid edema related to VHL disease, which, to the best of our knowledge, has never been reported previously. In spite of the short time during which the patient was observed, and although several other studies may be required for steroids to be confirmed as a valuable therapeutic strategy, we assume that, in combination with other therapies, the Ozurdex™ intravitreal implant may provide a potentially useful tool for the treatment of RHB-related macular edema in VHL disease.

## Abbreviations

BCVA: Best corrected visual acuity
DD: Disk diameter
ETDRS: Early Treatment Diabetic Retinopathy Study
HIF: Hypoxia-inducible factor
IDI: Intravitreal dexamethasone implant
IOP: Intraocular pressure
OCT: Optical coherence tomography
pVHL: von Hippel-Lindau protein
RE: Right eye
RHB: Retinal hemangioblastoma
SS-OCT: Swept-source optical coherence tomography
VEGF: Vascular endothelial growth factor
VHL: von Hippel-Lindau
